# Development and validation of a risk score nomogram model to predict the risk of 5-year all-cause mortality in diabetic patients with hypertension: A study based on NHANES data

**DOI:** 10.1016/j.ijcrp.2024.200265

**Published:** 2024-03-27

**Authors:** Hongzhao You, Dingyue Zhang, Yilu Liu, Yanyan Zhao, Ying Xiao, Xiaojue Li, Shijie You, Tianjie Wang, Tao Tian, Haobo Xu, Rui Zhang, Dong Liu, Jing Li, Jiansong Yuan, Weixian Yang

**Affiliations:** a Department of Cardiology, Fuwai Hospital, National Centre for Cardiovascular Diseases, National Clinical Research Centre for Cardiovascular Diseases, Chinese Academy of Medical Sciences and Peking Union Medical College, Beijing, China; b Endocrinology Centre, Fuwai Hospital, National Centre for Cardiovascular Diseases, Chinese Academy of Medical Sciences and Peking Union Medical College, Beijing, China; c Department of Internal Medicine, Peking Union Medical College Hospital, Chinese Academy of Medical Sciences and Peking Union Medical College, Beijing, China; d Medical Research and Biometrics Centre, National Centre for Cardiovascular Diseases, Beijing, China

**Keywords:** Diabetes, Hypertension, All-cause mortality, Nomogram, NHANES

## Abstract

**Background:**

The present study aimed to develop and validate a prediction nomogram model for 5-year all-cause mortality in diabetic patients with hypertension.

**Methods:**

Data were extracted from the National Health and Nutrition Examination Survey (NHANES). A total of 3291 diabetic patients with hypertension in the NHANES cycles for 1999–2014 were selected and randomly assigned at a ratio of 8:2 to the training cohort (n = 2633) and validation cohort (n = 658). Multivariable Cox regression was conducted to establish a visual nomogram model for predicting the risk of 5-year all-cause mortality. Receiver operating characteristic curves and C-indexes were used to evaluate the discriminant ability of the prediction nomogram model for all-cause mortality. Survival curves were created using the Kaplan–Meier method and compared by the log-rank test.

**Results:**

The nomogram model included eight independent predictors: age, sex, education status, marital status, smoking, serum albumin, blood urea nitrogen, and previous cardiovascular disease. The C-indexes for the model in the training and validation cohorts were 0.76 (95% confidence interval: 0.73–0.79, p < 0.001) and 0.75 (95% confidence interval: 0.69–0.81, p < 0.001), respectively. The calibration curves indicated that the model had satisfactory consistency in the two cohorts. The risk of all-cause mortality gradually increased as the tertiles of the nomogram model score increased (log-rank test, p < 0.001).

**Conclusion:**

The newly developed nomogram model, a readily useable and efficient tool to predict the risk of 5-year all-cause mortality in diabetic patients with hypertension, provides a novel risk stratification method for individualized intervention.

## Abbreviations

NHANES: National Health and Nutrition Examination Survey
DM: diabetes mellitus
ROC: receiver operator characteristic
CI: confidence interval
ASCVD: atherosclerotic cardiovascular disease
BMI: body mass index
SBP: systolic blood pressure
DBP: diastolic blood pressure
BUN: blood urea nitrogen

## 1 Introduction

As one of the most common metabolic diseases, diabetes mellitus is a serious global public health concern with a large economic burden [1]. The presence of diabetes can increase the risk for all types of macrovascular disease, including ischemic and hemorrhagic stroke [2], as well as microvascular complications in vital organs such as the kidneys (nephropathy), eyes (retinopathy), and nervous system (neuropathy). In addition to vascular complications, diabetes can elevate all-cause mortality by increasing the risk of infection, cancer, and chronic kidney disease [3]. The interaction of diabetes and hypertension and the resulting impact on the prognosis of diabetic patients are widely known, and an intensive pharmacological approach for this population is recommended in the current guidelines [4,5]. Blood glucose and blood pressure share several pathophysiological mechanisms that clarify the coexistence of diabetes and hypertension, including insulin resistance, obesity, increased oxidative stress, endothelial dysfunction, and inflammation [6]. It was demonstrated that the rates of both atherosclerotic cardiovascular disease (ASCVD)-related and all-cause mortality were significantly increased in diabetic patients with hypertension compared with non-hypertensive diabetic patients [7]. Thus, accurate prediction of mortality is essential for better management of both diabetes and hypertension. Although several traditional risk factors, such as obesity, renal diseases, and dyslipidemia, were found to be associated with ASCVD onset and ASCVD-related mortality in diabetic patients with hypertension [8,9], few of these factors could effectively predict all-cause mortality.

As visual statistical prognostic tools, nomogram models are widely used for clinical risk stratification and prediction of clinical outcomes by calculating scores for potential predictors [10]. In the present study, we developed and validated a prediction nomogram model for 5-year all-cause mortality in diabetic patients with hypertension based on data from the National Health and Nutrition Examination Survey (NHANES), a consecutive survey series on the health and nutrition status of the general population in the United States.

## 2 Materials and methods

### 2.1 Study design and participants

Data were extracted from the NHANES, a survey series with a complex, stratified, multistage sampling design that aims to determine the health status of citizens in the United States. All of the participants provided signed informed consent, and the survey protocols were approved by the Research Ethics Review Board of the National Center for Health Statistics. For the present study, we selected participants in the 1999–2014 cycles of the NHANES and divided them at a ratio of 8:2 into the training cohort and validation cohort. We included participants aged between 20 and 75 years with coexisting hypertension and diabetes at baseline. Hypertension was defined as self-reported hypertension, systolic blood pressure (SBP) ≥140 mmHg or diastolic blood pressure (DBP) ≥90 mmHg (average of three recorded measurements), or reported use of anti-hypertensive medication [11]. Diabetes was defined as fasting plasma glucose ≥7.0 mmol/L (126 mg/dL), hemoglobin A1c (HbA1c) > 6.5%, self-reported doctor-diagnosed type 2 diabetes, and/or use of insulin or oral hypoglycemic medication. Participants without information on follow-up outcomes and key candidate variables were excluded. A flowchart of the study is shown in Figure S1. All-cause mortality during follow-up was determined using the linked National Death Index through to December 31, 2019. The training and validation cohorts were selected to provide at least 5 years of follow-up for evaluation of all-cause mortality. All procedures were performed in accordance with relevant guidelines and regulations.

### 2.2 Potential predictors

We included various predictors for mortality in patients with hypertension or diabetes mellitus based on previous studies. The predictors included demographic characteristics (age, sex, ethnicity, education, marital status, income-to-poverty ratio, smoking), comorbidities (stroke, dyslipidemia, chronic kidney disease, myocardial infarction, cardiovascular disease), physical examination data (body mass index, waist circumference, SBP, DBP), and laboratory data (total cholesterol/high-density lipoprotein-cholesterol ratio, HbA1c, white blood cell count, hemoglobin, platelet count, albumin, blood urea nitrogen, blood uric acid, urine albumin/creatinine ratio, estimated glomerular filtration rate). Total cholesterol, triglyceride, low-density lipoprotein-cholesterol, and high-density lipoprotein-cholesterol concentrations were measured using a Hitachi 704 Analyzer. HbA1c measurements were performed using a Glycohemoglobin Analyzer. Serum creatinine and uric acid levels were detected using a Beckman automated clinical analyzer. Hemoglobin, white blood cell count, and platelet count were evaluated using automated hematology analyzing devices [10]. Albumin concentrations were measured by a bichromatic digital endpoint method using DxC800 modular chemistry. The blood urea nitrogen concentration was determined by an enzymatic conductivity rate method using DxC800 modular chemistry. Ethnicity was categorized into four groups (non-Hispanic White, non-Hispanic Black, Hispanic/Mexican, other races), education into two levels (less than high school, high school or above), marital status into three groups (married, widowed or divorced, single), and smoking into three groups (never, former, current). Cardiovascular disease included congestive heart failure, coronary heart disease, angina pectoris, heart attack, and stroke. The estimated glomerular filtration rate was calculated using the abbreviated CKD-EPI 2009 formula.

### 2.3 Statistical analysis

All statistical analyses were conducted using R version 4.0.3 statistical software (R Core Team Vienna, Austria).A two-sided value of p < 0.05 was considered statistically significant. Continuous variables were described as mean and standard deviation, and categorical variables were expressed as count and percentage. The hazard ratio (HR) and 95% confidence interval (CI) were estimated for all variables using a Cox regression model. To identify the final prediction model, a backward stepwise selection method with the Akaike information criterion was conducted to select the best potential predictive variables by multivariable Cox regression. The nomogram was depicted based on the final prediction model using the “rms” package. To test the model's performance, internal validation by bootstrapping with 1000 resamples and external validation were performed in the training cohort and validation cohort, respectively. The performance of the model was estimated by measuring the discrimination and calibration. Discrimination efficiency refers to the capacity of a prediction model to distinguish between patients with and without the outcome. Receiver operating characteristic curves and concordance indexes (C-indexes) were applied to evaluate the discrimination, with C-index ≥0.7 defined as good discrimination. Calibration refers to the agreement between the predicted outcomes and the actual outcomes. Calibration curves were depicted to evaluate the calibration, and curves close to the diagonal line were considered to indicate perfect calibration.

## 3 Results

### 3.1 Baseline characteristics

A total of 3291 patients were selected for the study, comprising 2633 participants in the training cohort and 658 participants in the validation cohort. During the follow-up period, 219 patients in the training cohort had all-cause death. The baseline characteristics of the patients in the training cohort are shown in Table S1. Compared with the surviving patients, the patients with all-cause death had several significant differences. Specifically, the patients with all-cause death were older (64.6 ± 8.2 years vs. 59.7 ± 10.5 years, p < 0.001) and less likely to be female (36.1% vs. 51.5%, p = 0.009). They had higher SBP (140.6 ± 21.8 mmHg vs. 136.4 ± 20.0 mmHg, p = 0.006). They also showed significant differences in marital status, smoking history, and race. The rates of former and current smoking history, as well as being single, widowed or divorced were higher in all-cause death group, comparing with survival group (all p value < 0.05). Additionally, a higher proportion of non-Hispanic white individuals were observed in this group. In terms of laboratory examination findings, the patients with all-cause death had lower hemoglobin (13.5 ± 1.8 g/dL vs. 13.9 ± 1.6 g/dL, p = 0.002) and serum albumin (39.9 ± 4.0 g/dL vs. 41.6 ± 3.2 g/dL, p < 0.001), but higher blood uric acid (379.4 ± 104.4 μmol/L vs. 345.0 ± 93.1 μmol/L, p < 0.001), blood urea nitrogen (7.7 ± 5.6 mg/dL vs. 5.5 ± 2.7 mg/dL, p < 0.001) and urinary microalbumin (117.1 ± 407.8 g/g Cr vs. 74.7 ± 497.9 g/g Cr, p < 0.001). The proportions for history of stroke (13.2% vs. 7.3%, p < 0.001), chronic kidney disease (77.2% vs. 58.7%, p < 0.001) and cardiovascular disease (44.3% vs. 24.1%, p < 0.001) were higher in the patients with all-cause death. The characteristics of the patients in the validation cohort were partly similar to those in the training cohort (Table S2).

### 3.2 Multivariable Cox regression analysis of independent predictors for 5-year all-cause mortality

To identify independent factors that can predict 5-year all-cause mortality in diabetic patients with hypertension, a multivariable Cox regression analysis was conducted. As shown in Table 1, eight predictors were independently associated with 5-year all-cause mortality: age (HR = 1.07, 95% CI: 1.05–1.10, p < 0.001), male sex (HR = 1.77, 95% CI: 1.21–2.58, p = 0.003), higher education status (HR = 0.70, 95% CI: 0.42–0.96, p = 0.034), marital status (widowed or divorced, HR = 1.84, 95% CI: 1.20–2.82, p = 0.006; single, HR = 2.37, 95% CI: 1.42–3.98, p = 0.001), smoking (former, HR = 1.58, 95% CI: 1.04–2.42, p = 0.034; current, HR = 1.92, 95%CI: 1.12–3.33, p = 0.021), serum albumin (HR = 0.86, 95% CI: 0.82–0.90, p < 0.001), blood urea nitrogen (HR = 1.10, 95%CI: 1.04–1.16, p < 0.001), and previous cardiovascular disease (HR = 1.41, 95% CI: 1.00–1.98, p = 0.049).

**Table 1:** Univariable and multivariable Cox analyses for factors predicting 5-year mortality of diabetic patients with hypertension in the training cohort.

Index	Univariable		Multivariable	
	HR and 95%CI	P value	HR and 95%CI	P value
Age	1.05 (1.03–1.08)	<0.001	1.07 (1.05–1.10)	<0.001
Sex				
Female	Ref.			
Male	1.46 (1.04–2.06)	0.029	1.77 (1.21–2.58)	0.003
Education				
Less than high-school	Ref.			
High school or above	0.71 (0.51–0.99)	0.043	0.70 (0.42–0.96)	0.037
Marital Status				
Married				
Widowed or divorced	1.63 (1.03–2.58)	0.038	1.84 (1.20–2.82)	0.006
Single	1.98 (1.36–2.89)	<0.001	2.37 (1.42–3.98)	0.001
Smoking				
Never				
Former	2.07 (1.28–3.35)	0.003	1.58 (1.04–2.42)	0.034
Current	2.09 (1.42–3.06)	<0.001	1.92 (1.12–3.33)	0.021
Physical activity				
No exercise	Ref.	0.136		
Moderate intensity	1.04 (0.98–1.10)			
Vigorous intensity	1.12 (0.99–1.21)			
Alcohol use				
No	Ref.			
Yes	1.10 (0.89–1.22)	0.321		
BMI	0.98 (0.95–1.01)	0.110		
SBP	1.01 (1.01–1.02)	<0.001		
DBP	1.00 (0.99–1.01)	0.655		
White blood cell	1.08 (1.01–1.16)	0.021		
Hemoglobin	0.82 (0.75–0.91)	<0.001		
Platelet	1.00 (0.99–1.00)	0.880		
Albumin	0.86 (0.82–0.90)	<0.001	0.86 (0.82–0.90)	<0.001
Blood urea nitrogen,	1.16 (1.11–1.21)	<0.001	1.10 (1.04–1.16)	<0.001
Blood uric acid	1.00 (1.00–1.01)	0.009		
eGFR	0.98 (0.97–0.98)	<0.001		
TC/HDL	0.92 (0.83–1.03)	0.141		
HbAlc	0.99 (0.90–1.09)	0.771		
UACR,	1.00 (0.99–1.00)	0.539		
Stroke	0.44 (0.28–0.70)	<0.001		
Dyslipidemia	2.77 (1.97–3.89)	<0.001		
Chronic Kidney Disease	2.28 (1.54–3.38)	<0.001		
Cardiovascular Diseases	0.91 (0.62–1.33)	0.615	1.41 (1.00–1.98)	0.049

BMI, body mass index; SBP, systolic blood pressure; DBP, diastolic blood pressure; eGFR, estimated glomerular filtration rate; HbAlc, glycosylated hemoglobin; TC, total cholesterol; HDL, high-density lipoprotein; UACR, urinary albumin/creatinine ratio.

### 3.3 Development and validation of the nomogram model

**Fig. 1:**
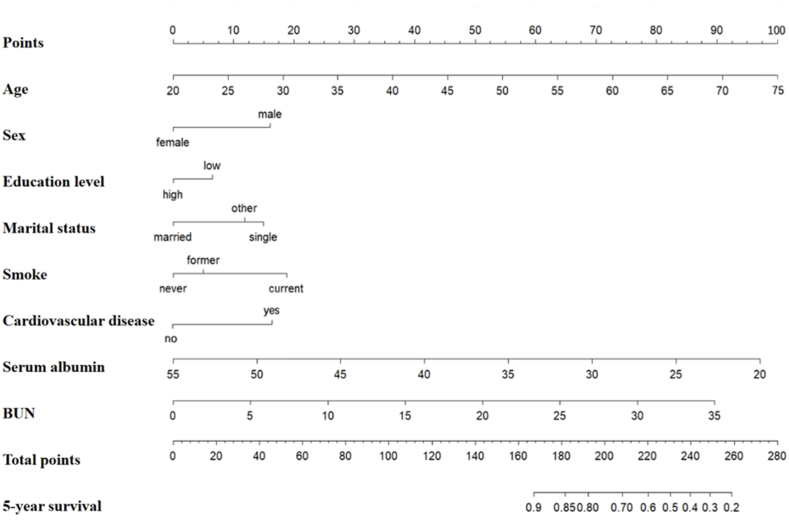
Nomogram for estimating the 5-year all-cause mortality probability in diabetic patients with hypertension. BUN, blood urea nitrogen.

**Fig. 2:**
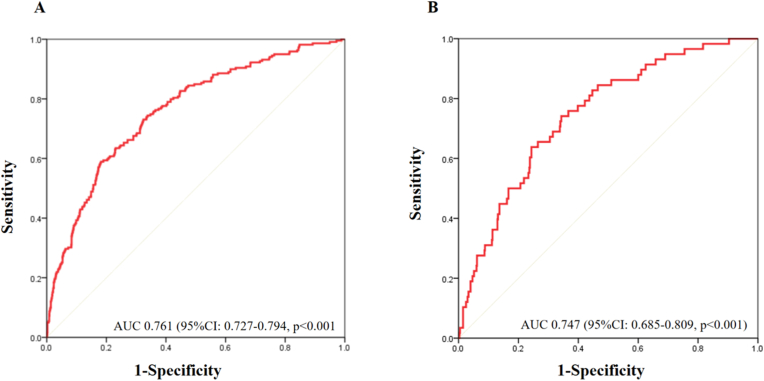
The area under the receiver operating characteristic (ROC) curves of the nomogram for predicting 1-year all-cause mortality in training cohort (A) and validation cohort (B).

We constructed a nomogram model to predict the risk of 5-year all-cause mortality in diabetic patients with hypertension, based on the multivariable Cox regression analysis and clinical practice. As shown in Fig. 1, the probability of 5-year all-cause mortality could be estimated by calculating the total number of points from a vertical line for each variable to the point axis. Among the eight selected predictors, age had the strongest impact on patient prognosis, followed by blood urea nitrogen and serum albumin. Consistent with the results of the receiver operating characteristic curve analysis, the C-indexes for the training and validation cohorts were 0.76 (95%CI: 0.73–0.79, p < 0.001) and 0.75 (95%CI: 0.69–0.81, p < 0.001), indicating that the nomogram model had good performance for discriminating 5-year all-cause mortality (Fig. 2A and B). The calibration plots showed that the predictions of the nomogram model for the risk of all-cause mortality were highly consistent with the actual observations (Figure S2).

### 3.4 Prognostic value of the nomogram model

**Fig. 3:**
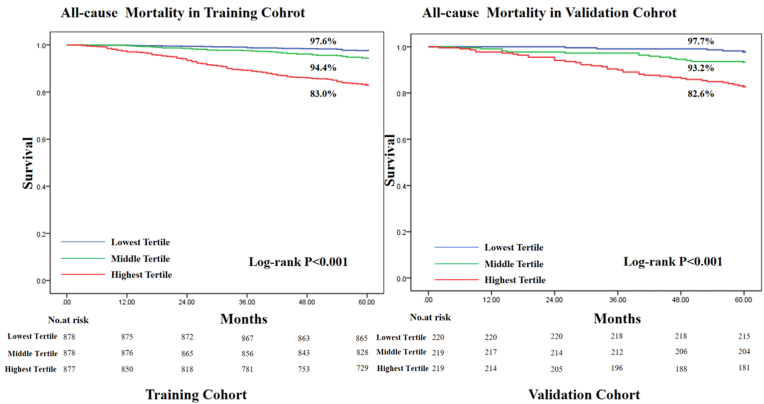
Kaplan-Meier survival curves of diabetic patients with hypertension ranked by tertiles of the score of the nomogram model.

We named the score of nomgram model as DHAM (“Diabetic patient with Hypertension All-cause Death”) score. The patients were divided according to the tertiles of DHAM score. Compared with the lowest tertile group, the highest tertile group had higher rates of all-cause death in both the training cohort (16.9% vs 1.5%, p < 0.001) and the validation cohort (17.4% vs. 2.2%, p < 0.001) (Figure S3). We further plotted Kaplan–Meier survival curves for the patients in the low, middle, and high tertile groups (Fig. 3). The risk of all-cause mortality gradually increased as the tertiles of the DHAM score, indicating that the nomogram model had a robust prognostic value (log-rank test, p < 0.001).

## 4 Discussion

In the present study, we developed and validated a new nomogram model containing eight identified predictive factors to predict the risk of 5-year all-cause mortality in diabetic patients with hypertension. The prediction nomogram model had an excellent prognostic value and allowed more precise risk stratification for diabetic patients with hypertension.

Hypertension and diabetes mellitus are both major global public health problems, and their frequent coexistence results in significant morbidity and mortality. It is believed that high blood pressure can increase the risks of both all-cause death [12] and cardiovascular death [13] in patients with diabetes, and the current guidelines recommend comprehensive management of risk factors to reduce the risk of cardiovascular events [14]. Few previous studies have focused on identifying predictors of all-cause death, and an individualized and visual risk calculator for all-cause mortality was not provided. The lack of a risk stratification method for all-cause mortality is an obstacle for physicians regarding intensive intervention.

Nomogram models have been widely applied for the prognosis of various cardiovascular diseases in recent years. To effectively predict the risk of all-cause mortality in diabetic patients with hypertension, we developed a prognostic nomogram model based on NHANES data. Eight available predictors for 5-year mortality, including age, sex, education, marital status, smoke, SBP, blood urea nitrogen, and cardiovascular disease, were identified by the Cox regression model and used to construct the nomogram model. Among these predictors, age, sex, and history of cardiovascular disease have been recognized as risk factors for all-cause mortality in patients with diabetes [15]. Age has been demonstrated to be positively associated with both ASCVD-related and all-cause mortality [16] in people with diabetes, indicating that targeted anti-aging therapies may have potential to halt the progression of diabetes [17]. Both uncontrolled SBP and smoking are established risk factors for all-cause mortality in patients with diabetes [18]. A healthy lifestyle can significantly reduce the risk of all-cause mortality. Our findings indicated that long-term lifestyle management, including anti-hypertensive medication and smoking cessation, may be beneficial in diabetic patients with hypertension. Several studies have demonstrated significant associations between demographic variables and prognosis in patients with diabetes, especially socioeconomic variables. In the present study, two demographic variables, marital status and education level, were identified as parameters that can predict the risk of 5-year all-cause mortality, indicating that social support may play an important role in the clinical outcomes of both diabetes and hypertension. In a population-based study, Slåtsve et al. [19]. demonstrated that higher education levels were associated with lower odds for coronary heart disease and chronic kidney disease in individuals with type 2 diabetes, and higher education was also found to affect their prognosis. The associations between marital status and health outcomes varied by sex. It was reported that the social support offered by marriage exerts a protective effect for men, but not for women [20]. However, few studies have focused on how these social factors affect the prognosis of patients with coexisting hypertension and diabetes. The present study provides a new view regarding social support for diabetes and hypertension, and more studies in this field are warranted. As the strongest biomarker predictor impacting patient prognosis in our nomogram model, serum albumin is a predominant protein in human plasma. It plays an important role in the maintenance of homeostasis, creates a balance between hydrostatic and colloid osmotic pressure within vessels, and also contributes to the progression of cardiovascular diseases with its anti-inflammatory, antioxidant, anticoagulant, and anti-platelet aggregation activities [21]. Given these biological functions, serum albumin has attracted considerable attention for prediction of survival in diabetic patients in previous studies [22]. Our study demonstrated that hypoalbuminemia may be an independent predictor for all-cause mortality in diabetic patients with hypertension. Blood urea nitrogen is a protein metabolic waste product produced by the liver and excreted by the kidneys and is associated with an increased risk of incident diabetes [23]. In a recent study involving a NHANES cohort, Hong et al. found that blood urea nitrogen was an independent predictor for both cardiovascular disease-related and all-cause mortality in US adults [24]. The eight predictors in our model included a wide range of demographic characteristics, physical examination findings, and laboratory examination data, and thus the DHAM score has the potential to guide comprehensive risk factor management and assist with decision-making on medical therapy, social support, and public health.

Our study is the first to construct and validate a prognostic nomogram model for the risk of 5-year mortality in diabetic patients with hypertension. Diabetic patients with hypertension have traditionally been identified as a population at high risk of cardiovascular events and all-cause death [25]. However, few studies have focused on precise risk stratification in this population. Our scoring system provides a reliable and propagable prediction model compared with other nomogram models, and can predict the risk of all-cause death. The patients were further classified into low-, middle-, and high-risk subgroups according to the tertiles of DHAM score. Hence, the newly developed nomogram model can discriminate the patients at high risk of all-cause mortality among diabetic patients with hypertension and has the potential to provide a new approach for clinical decision-making regarding aggressive intervention.

There were several limitations to the study. First, NHANES is a multisatge, national area probability survey, with intricate sample designs and sample sizes. Our data from NHANES were not weighted [26], and it might affect the construction of prediction model. Therefore, external validation is required to confirm whether the prognostic model is suitable for different populations Second, the laboratory examination data were extracted from medical records, and thus some novel biomarkers that can predict all-cause mortality, such as interleukin-6, homocysteine, and low-density lipoprotein-cholesterol, were not provided. Third, we utilized NHANES data from 1999 to 2014 as a complete 5-year follow-up period. To validate the prognostic value of our prediction model and perform subgroup analyses, updated data for a larger and renewed population should be employed in future studies. Despite these limitations, a predictive nomogram model was constructed that can act as a reliable and accurate risk stratification tool for all-cause mortality in diabetic patients with hypertension.

## 5 Conclusions

The newly developed and validated nomogram model is a robust tool for predicting the risk of 5-year all-cause mortality in diabetic patients with hypertension, and provides a novel approach to risk stratification in this special population.

## Ethics approval and consent to participate

The study was designed and carried out following the principles of the Declaration of Helsinki. The study was approved by the Fuwai Hospital Ethics Committee.

## Consent for publication

Not applicable.

## Availability of data and materials

The data that support the findings of this study are available from the corresponding author upon reasonable request.

## Competing interests

All patients have no conflicts of interest to disclose.

## Funding

This work was supported by the Youth Science Foundation of Fuwai's Hospital, Chinese Academy of Medical Science (Grant NO. 2022-FWQN08) and 10.13039/501100001809National Natural Science Foundation of China (Grant NO. 82192902).

## CRediT authorship contribution statement

**Hongzhao You:** Writing – original draft, Visualization, Funding acquisition, Conceptualization. **Dingyue Zhang:** Writing – review & editing, Formal analysis. **Yilu Liu:** Data curation. **Yanyan Zhao:** Validation, Methodology, Conceptualization. **Ying Xiao:** Resources. **Xiaojue Li:** Conceptualization. **Shijie You:** Supervision. **Tianjie Wang:** Methodology. **Tao Tian:** Methodology. **Haobo Xu:** Visualization. **Rui Zhang:** Investigation. **Dong Liu:** Validation. **Jing Li:** Supervision. **Jiansong Yuan:** Project administration. **Weixian Yang:** Funding acquisition.

## References

[1] Bommer C., Heesemann E., Sagalova V. (2017 Jun). The global economic burden of diabetes in adults aged 20-79 years: a cost-of-illness study. Lancet Diabetes Endocrinol..

[2] Sarwar N., Gao P., Seshasai S.R. (2010 Jun 26). Diabetes mellitus, fasting blood glucose concentration, and risk of vascular disease: a collaborative meta-analysis of 102 prospective studies. Lancet.

[3] Ma R.C.W. (2018 Jun). Epidemiology of diabetes and diabetic complications in China. Diabetologia.

[4] ElSayed N.A., Aleppo G., Aroda V.R. (2023 Jan 1). Cardiovascular disease and risk management: Standards of Care in diabetes-2023. Diabetes Care.

[5] Marx N., Federici M., Schütt K. (2023 Oct 14). 2023 ESC Guidelines for the management of cardiovascular disease in patients with diabetes. Eur. Heart J..

[6] Colussi G., Da Porto A., Cavarape A. (2020 Feb). Hypertension and type 2 diabetes: lights and shadows about causality. J. Hum. Hypertens..

[7] Sabuncu T., Sonmez A., Eren M.A. (2021 Apr). Characteristics of patients with hypertension in a population with type 2 diabetes mellitus. Results from the Turkish Nationwide SurvEy of Glycemic and other metabolic parameters of patients with diabetes mellitus (TEMD hypertension study). Prim Care Diabetes.

[8] Campbell N.R., Leiter L.A., Larochelle P. (2009 May). Hypertension in diabetes: a call to action. Can. J. Cardiol..

[9] Toto R.D. (2007 Nov). Reducing cardiovascular events in high-risk patients: the challenge of managing hypertension in patients with diabetic renal disease. J. Clin. Hypertens..

[10] Zhang H., Tian W., Sun Y. (2022 May 4). Development, validation, and visualization of a web-based nomogram to predict 5-year mortality risk in older adults with hypertension. BMC Geriatr..

[11] Mancia G., Kreutz R., Brunström M. (2023 Jun 21). 2023 ESH guidelines for the management of arterial hypertension the Task Force for the management of arterial hypertension of the European Society of hypertension Endorsed by the International Society of hypertension (ISH) and the European renal association (ERA). J. Hypertens..

[12] Al-Rubeaan K., Youssef A.M., Ibrahim H.M. (2016 Aug). All-cause mortality and its risk factors among type 1 and type 2 diabetes mellitus in a country facing diabetes epidemic. Diabetes Res. Clin. Pract..

[13] Yun J.S., Ko S.H. (2021 Oct). Current trends in epidemiology of cardiovascular disease and cardiovascular risk management in type 2 diabetes. Metabolism.

[14] American Diabetes Association Professional Practice Committee (2024 Jan 1). 10. Cardiovascular disease and risk management: Standards of Care in diabetes-2024. Diabetes Care.

[15] Kristensen S.L., Rørth R., Jhund P.S. (2019). Cardiovascular, mortality, and kidney outcomes with GLP-1 receptor agonists in patients with type 2 diabetes: a systematic review and meta-analysis of cardiovascular outcome trials. Lancet Diabetes Endocrinol..

[16] Yang J.J., Yu D., Wen W. (2019 Apr 5). Association of diabetes with all-cause and cause-specific mortality in Asia: a Pooled analysis of more than 1 Million participants. JAMA Netw. Open.

[17] Chen L., Yin X., Zhao Y., Chen H., Tan T., Yao P., Tang Y. (2022 Sep). Biological ageing and the risks of all-cause and cause-specific mortality among people with diabetes: a prospective cohort study. J. Epidemiol. Community Health.

[18] Han H., Cao Y., Feng C. (2022 Feb 1). Association of a healthy lifestyle with all-cause and cause-specific mortality among individuals with type 2 diabetes: a prospective study in UK Biobank. Diabetes Care.

[19] Slåtsve K.B., Claudi T., Lappegård K.T. (2022 Sep). Level of education is associated with coronary heart disease and chronic kidney disease in individuals with type 2 diabetes: a population-based study. BMJ Open Diabetes Res Care.

[20] Ramezankhani A., Azizi F., Hadaegh F. (2019 Apr 22). Associations of marital status with diabetes, hypertension, cardiovascular disease and all-cause mortality: a long term follow-up study. PLoS One.

[21] Zhu L., Chen M., Lin X. (2020 Jan 31). Serum albumin level for prediction of all-cause mortality in acute coronary syndrome patients: a meta-analysis. Biosci. Rep..

[22] Zhang J., Zhang R., Wang Y. (2019 Feb 17). The level of serum albumin is associated with renal prognosis in patients with diabetic nephropathy. J. Diabetes Res..

[23] Xie Y., Bowe B., Li T. (2018 Mar). Higher blood urea nitrogen is associated with increased risk of incident diabetes mellitus. Kidney Int..

[24] Hong C., Zhu H., Zhou X. (2023 Jan 16). Association of blood urea nitrogen with cardiovascular diseases and all-cause mortality in USA adults: results from NHANES 1999-2006. Nutrients.

[25] Horr S., Nissen S. (2016 Jun). Managing hypertension in type 2 diabetes mellitus. Best Pract Res Clin Endocrinol Metab.

[26] Yang L., Shen X., Seyiti Z. (2023 Nov 22). Development and validation of a nomogram for predicting all-cause mortality in American adult hypertensive populations. Front. Pharmacol..

