# Supplementary material for: Development and validation of a risk score nomogram model to predict the risk of 5-year all-cause mortality in diabetic patients with hypertension: A study based on NHANES data
Source: Int J Cardiol Cardiovasc Risk Prev. 2024 Mar 27;21:200265. doi: 10.1016/j.ijcrp.2024.200265 (PMC10992723; doi:10.1016/j.ijcrp.2024.200265)
Supplement: Multimedia component 1 [file mmc1.docx]

**Development and validation of a risk score nomogram model to predict the risk of 5-year all-cause mortality in diabetic patients with hypertension: a study based on NHANES data**

Hongzhao You ^a,b,c*#^, [Dingyue Zhang](https://pubmed.ncbi.nlm.nih.gov/?term=Hou+X&cauthor_id=35306045) ^c*^, [Y](https://pubmed.ncbi.nlm.nih.gov/?term=Zhang+H&cauthor_id=35306045)ilu Liu ^a^, Yanyan Zhao ^d^, Ying Xiao^a^, Xiaojue Li ^b^, Shijie You ^a^, Tianjie Wang ^a^,Tao Tian ^a^, Haobo Xu ^a^, Rui Zhang ^b^, Dong Liu^a^, Jing Li ^c^, Jiansong Yuan^a#^,Weixian Yang^a#^.

*a Department of Cardiology, Fuwai Hospital, National Centre for Cardiovascular Diseases, National Clinical Research Centre for Cardiovascular Diseases, Chinese Academy of Medical Sciences and Peking Union Medical College, Beijing, China.*

*b Endocrinology Center, Fuwai Hospital, National Center for Cardiovascular Diseases, Chinese Academy of Medical Sciences and Peking Union Medical College, Beijing, China.*

*c Department of Internal Medicine, Peking Union Medical College Hospital, Chinese Academy of Medical Sciences and Peking Union Medical College, Beijing, China.*

*d Medical Research and Biometrics Centre, National Centre for Cardiovascular Diseases, Beijing, China.*

***Hongzhao You and Dingyue Zhang contributed equally to this work.**

#**Address for correspondence：**

Dr. Hongzhao You : hongzhao_you@163.com

Prof. Jiansong Yuan: jsyuantg@163.com

Prof. Weixian Yang:ywx66@aliyun.com

**Table S1.** Baseline characteristics of the patients in the training cohort

| Variable | All Patients  (n=2633) | Survival  (n=2414) | All-cause Death (n= 219) | p Value* |
| --- | --- | --- | --- | --- |
| **[Demographic](javascript:;) Data** |  |  |  |  |
| Age,Yrs | 60.0±10.4 | 59.7±10.5 | 64.5±8.2 | **<0.001** |
| Sex (Female,%) | 788 (49.8) | 1244 (51.5) | 79 (36.1) | **0.009** |
| Ethnicity, n (%) |  |  |  | **0.026** |
| Non-Hispanic White | 879 (33.4) | 794 (32.9) | 85 (38.8) |  |
| Non-Hispanic Black | 826 (31.4) | 749 (31.0) | 77 (35.2) |  |
| Hispanic Mexican | 518 (19.7) | 484 (20.1) | 34 (15.5) |  |
| Other races | 410 (15.5) | 387 (16.0) | 23 (10.5) |  |
| Education, n (%) |  |  |  | 0.218 |
| less than high-school | 1004 (38.1) | 912 (37.8) | 92 (42.0) |  |
| high school or above | 1629 (61.9) | 1502 (62.2) | 127 (58.0) |  |
| Marital status, n (%) |  |  |  | **<0.001** |
| Married | 707 (26.9) | 628 (26.1) | 79 (36.1) |  |
| Widowed or divorced | 1485 (56.4) | 1389 (57.5) | 96 (43.8) |  |
| Single | 441 (16.7) | 397 (16.4) | 44 (20.1) |  |
| Smoking (n, %) |  |  |  | **<0.001** |
| Never | 470 (17.9) | 397 (16.4) | 73 (33.3) |  |
| Former | 1500 (57.0) | 1389 (57.5) | 111 (50.7) |  |
| Current | 663 (25.1) | 628 (26.1) | 35 (16.0) |  |
| Physical activity (n, %) |  |  |  | 0.125 |
| No exercise | 266 (10.1) | 248 (16.3) | 18 (8.1) |  |
| Moderate intensity | 1693 (64.3) | 1553 (64.3) | 140 (63.9) |  |
| Vigorous intensity | 674 (25.6) | 613 (19.4) | 61 (24.0) |  |
| Alcohol use (n, %) |  |  |  |  |
| No | 1123 (42.6) | 997 (41.3) | 94 (43.0) |  |
| Yes | 1510 (57.4) | 1417 (48.7) | 125 (57.0) |  |
| **Physical Examinations** |  |  |  |  |
| BMI, kg/m2 | 33.1±7.1 | 33.2±7.1 | 33.0±8.3 | 0.283 |
| Waist, cm | 110.8±15.2 | 110.6±15.1 | 112.6±16.1 | 0.110 |
| SBP, mmHg | 136.7±20.6 | 136.4±20.0 | 140.6±21.8 | 0.006 |
| DBP,mmHg | 71.4±15.8 | 71.5±15.9 | 70.5±15.2 | 0.159 |
| **Laboratory Data** |  |  |  |  |
| White blood cells, 1000 cells/uL | 7.4±2.5 | 7.5±2.1 | 7.5±2.5 | 0.847 |
| Hemoglobin, g/dL | 13.8±1.7 | 13.9±1.6 | 13.5±1.8 | **0.002** |
| Platelet, 1000 cells/uL | 249.7±74.5 | 250.0±71.5 | 246.8±102.1 | 0.254 |
| Albumin, g/dL | 41.5±3.3 | 41.6±3.2 | 39.9±4.0 | **<0.001** |
| Blood urea nitrogen, mg/dL | 5.7±3.1 | 5.5±2.7 | 7.7±5.6 | **<0.001** |
| Blood uric acid, umol/L | 347.3±94.5 | 345.0±93.1 | 379.4±104.4 | **<0.001** |
| eGFR, mg/min/1.73m2 | 81.1±25.6 | 82.1±24.3 | 65.6±29.1 | **<0.001** |
| TC/HDL | 4.3±1.6 | 4.3±1.6 | 4.3±1.5 | 0.990 |
| HbAlc,% | 7.4±1.8 | 7.4±1.7 | 7.5±2.1 | 0.558 |
| UACR, mg/g Cr | 78.2±491.4 | 74.7±497.9 | 117.1±407.8 | **<0.001** |
| **Comorbidities** |  |  |  |  |
| Stroke (%) | 146(9.2) | 177 (7.3) | 29 (13.2) | **<0.001** |
| Dyslipidemia (%) | 1996 (75.8) | 1835 (76.0) | 162 (74.0) | 0.499 |
| Chronic Kidney Disease (%) | 963(60.9) | 1416 (58.7) | 169 (77.2) | **<0.001** |
| Cardiovascular Diseases (%) | 459(29.0) | 581(24.1) | 97 (44.3) | **<0.001** |
| Data are shown as mean ± standard deviation.  BMI, body mass index; SBP, systolic blood pressure; DBP, diastolic blood pressure; eGFR, estimated glomerular filtration rate; HbAlc, glycosylated hemoglobin; TC, total cholesterol; HDL, high-density lipoprotein; UACR, urinary albumin/creatinine ratio.  *p<0.05, significant difference. | | | | |

**Table S2**. Baseline characteristics of the patients in the validation cohort

| Variable | All Patients  (n=961) | Survival  (n=883) | All-cause Death (n= 78) | p Value* |
| --- | --- | --- | --- | --- |
| **[Demographic](javascript:;) Data** |  |  |  |  |
| Age,Yrs | 58.9±10.3 | 58.4±10.4 | 63.6±7.2 | <0.001 |
| Sex (Female,%) | 452 (47.0) | 428 (48.5) | 24 (30.8) | 0.003 |
| Ethnicity, n (%) |  |  |  | 0.080 |
| Non-Hispanic White | 497(31.4) | 451(31.1) | 46(34.8) |  |
| Non-Hispanic Black | 517(32.7) | 465(32.1) | 52(39.4) |  |
| Hispanic Mexican | 203(12.8) | 192(13.3) | 11(8.3) |  |
| Other races | 364(23.0) | 341(23.5) | 23(17.4) |  |
| Education, n (%) |  |  |  | 0.351 |
| less than high-school | 304 (31.6) | 283(32.0) | 21 (26.9) |  |
| high school or above | 657 (68.4) | 600 (60.0) | 57 (73.1) |  |
| Marital status, n (%) |  |  |  | <0.001 |
| Married | 866(54.8) | 813(56.1) | 53(40.2) |  |
| Widowed or divorced | 410 (25.9) | 353(24.4) | 57(43.2) |  |
| Single | 305(19.3) | 283(19.5) | 22(16.7) |  |
| Smoking (n, %) |  |  |  | <0.001 |
| Never | 474 (49.3) | 453 (51.3) | 21 (26.9) |  |
| Former | 351 (36.5) | 313 (35.4) | 38 (48.7) |  |
| Current | 136 (14.2) | 117 (13.3) | 19 (24.4) |  |
| Physical activity (n, %) |  |  |  | 0.125 |
| No exercise | 103 (10.7) | 92 (10.4) | 11 (8.1) |  |
| Moderate intensity | 613 (63.8) | 567 (64.2) | 46 (63.9) |  |
| Vigorous intensity | 245 (25.5) | 224 (25.4) | 21 (24.0) |  |
| Alcohol use (n, %) |  |  |  |  |
| No | 424 (44.1) | 391 (44.3) | 33 (42.3) |  |
| Yes | 537 (55.9) | 492 (55.7) | 45 (57.7) |  |
| **Physical Examinations** |  |  |  |  |
| BMI, kg/m2 | 33.7±7.6 | 33.7±7.5 | 33.4±7.9 | 0.981 |
| Waist, cm | 112.3±16.8 | 112.1±16.5 | 115.6±19.6 | 0.072 |
| SBP, mmHg | 135.1±18.6 | 134.8±18.4 | 138.2±21.0 | 0.125 |
| DBP,mmHg | 73.7±13.3 | 73.9±13.3 | 72.1±12.4 | 0.264 |
| **Laboratory Data** |  |  |  |  |
| White blood cells, 1000 cells/uL | 7.5±2.3 | 7.4±2.3 | 7.7±2.4 | 0.299 |
| Hemoglobin, g/dL | 13.8±1.5 | 13.8±1.5 | 13.5±1.9 | 0.224 |
| Platelet, 1000 cells/uL | 236.2±71.3 | 235.3±67.9 | 246.0±101.4 | 0.592 |
| Albumin, g/dL | 41.5±3.2 | 41.7±3.2 | 40.±3.3 | <0.001 |
| Blood urea nitrogen, mg/dL | 5.6±2.9 | 5.5±2.8 | 7.0±3.1 | <0.001 |
| Blood uric acid | 351.0±93.5 | 347.5±92.8 | 391.2±91.5 | <0.001 |
| eGFR, mg/min/1.73m2 | 81.1±26.5 | 82.6±25.6 | 64.1±30.6 | <0.001 |
| TC/HDL | 4.5±1.6 | 4.4±1.6 | 4.7±1.7 | 0.201 |
| HbAlc,% | 7.4±1.7 | 7.4±1.7 | 8.0±1.9 | 0.001 |
| UACR, mg/g Cr | 174.3±611.7 | 150.8±540.2 | 440.3±1115.7 | <0.001 |
| **Comorbidities** |  |  |  |  |
| Stroke (%) | 70(7.3) | 59(6.7) | 11(14.1) | 0.016 |
| Dyslipidemia (%) | 668(69.5) | 615(69.6) | 53(67.9) | 0.755 |
| Chronic Kidney Disease (%) | 560 (58.3) | 501(56.7) | 59(75.6) | 0.01 |
| Cardiovascular Diseases (%) | 227 (23.6) | 193(21.9) | 34(43.6) | <0.001 |
| Data are shown as mean ± standard deviation.  BMI, body mass index; SBP, systolic blood pressure; DBP, diastolic blood pressure; eGFR, estimated glomerular filtration rate; HbAlc, glycosylated hemoglobin; TC, total cholesterol; HDL, high-density lipoprotein; UACR, urinary albumin/creatinine ratio.  *p<0.05, significant difference. | | | | |

**
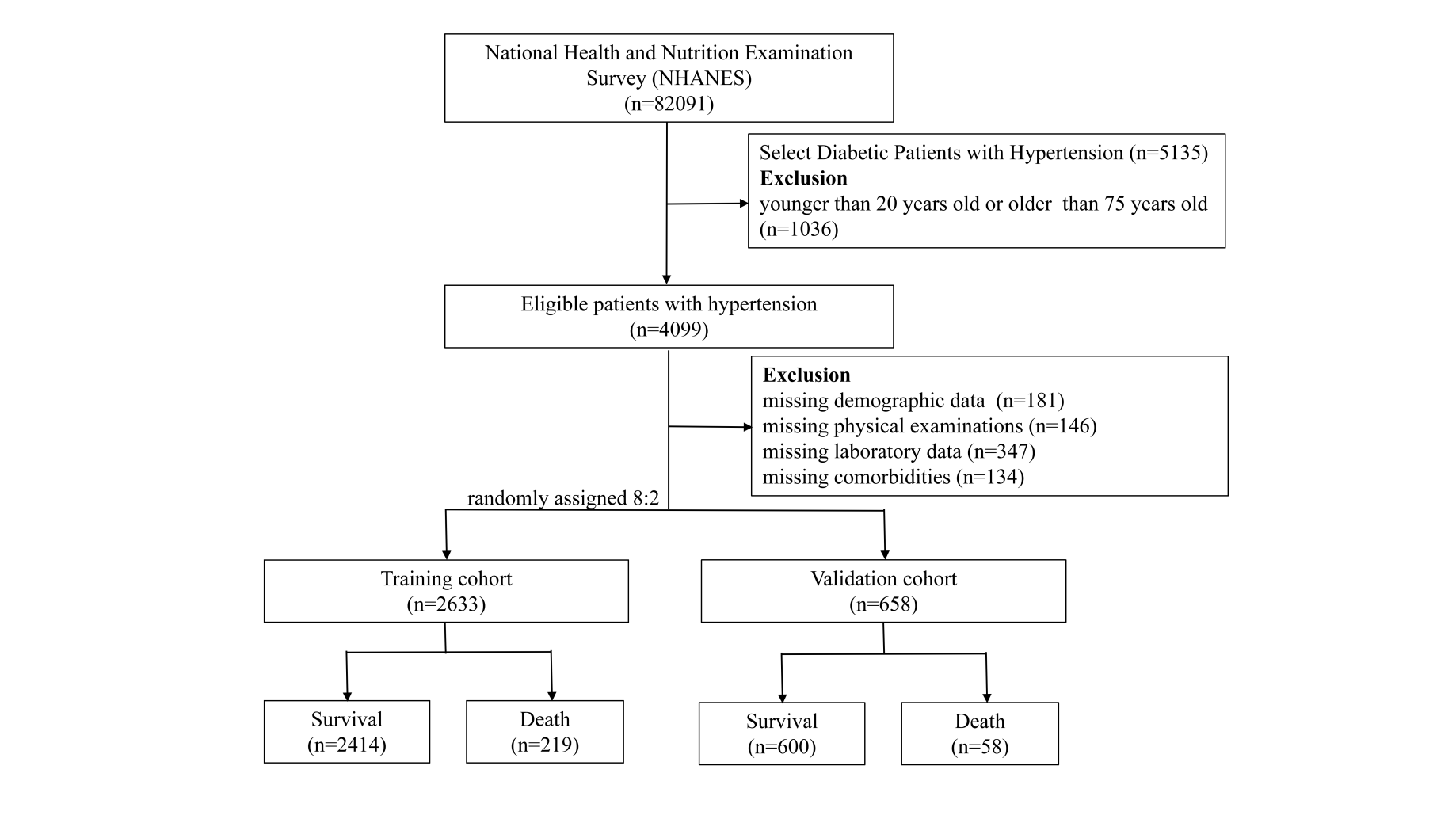
**

**Figure S1.** Flow chart of the study

**A**


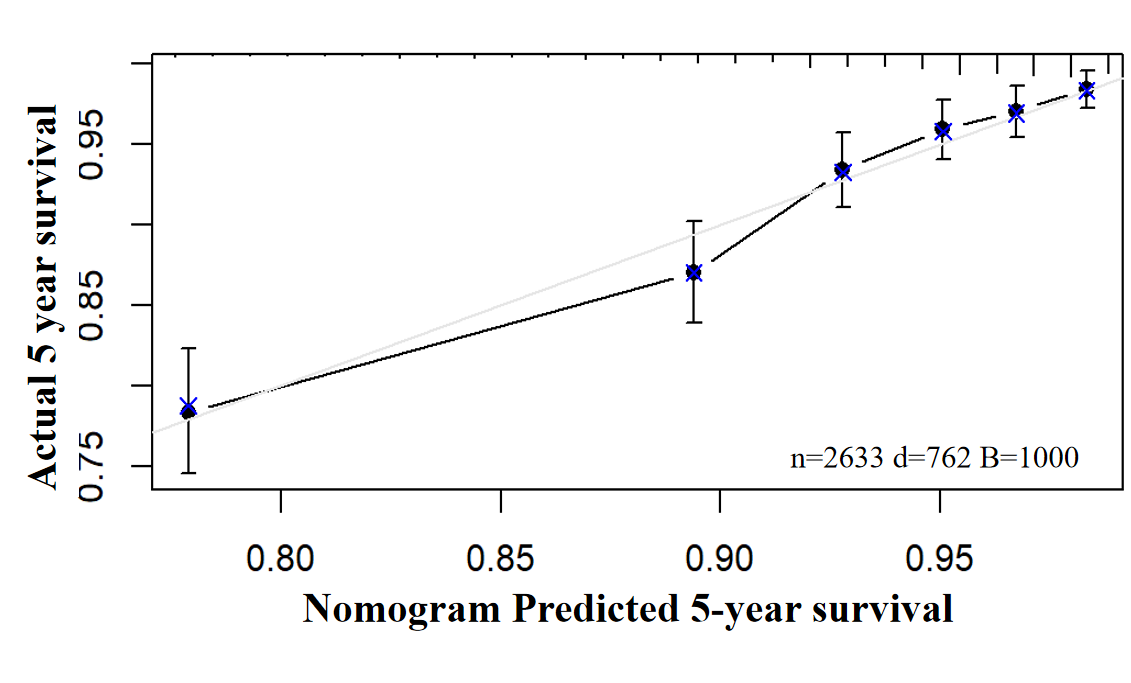


**B**

**
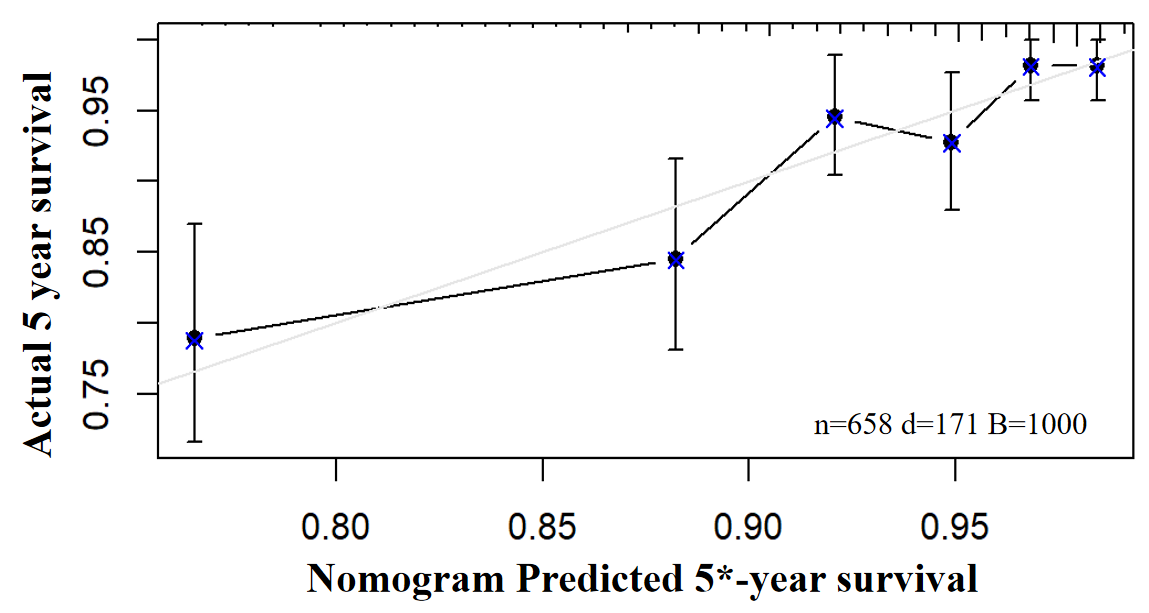
**

**Figure S2.** Calibration curves for the monogram in cohorts. A Training cohort. B validation cohort. The x- and y- axis represents the predicted probability and the actual observed probability of 5-year all-cause mortality, respectively.


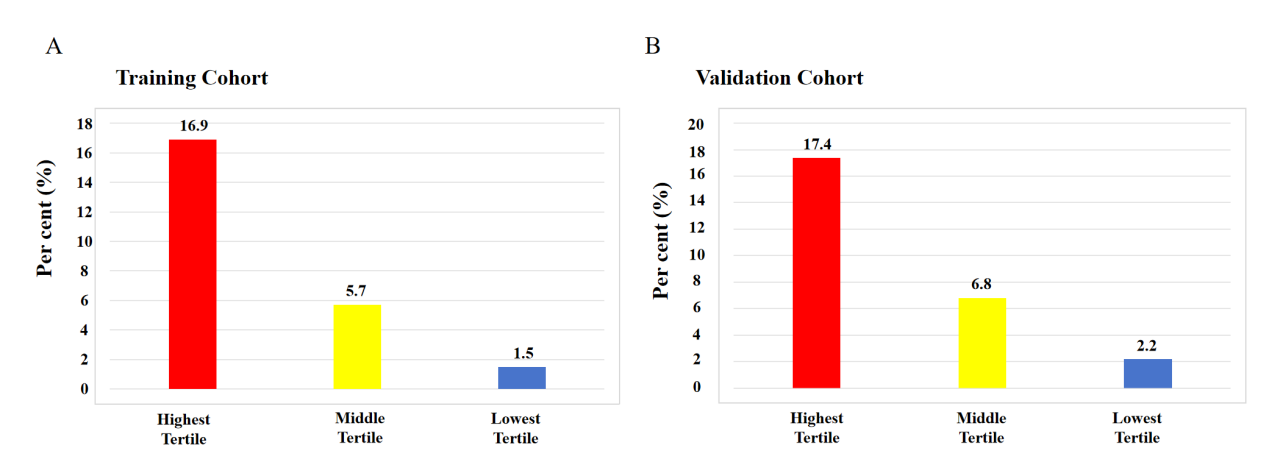


**Figure S3**. 5-year mortality rates of diabetic patients with hypertension by the tertiles of score of nomogram model. A,training cohort, B,validation cohort
